# Encapsulation-Stabilized, Europium Containing Nanoparticle as a Probe for Time-Resolved luminescence Detection of Cardiac Troponin I

**DOI:** 10.3390/bios7040048

**Published:** 2017-10-18

**Authors:** Ka Ram Kim, Yong Duk Han, Hyeong Jin Chun, Kyung Won Lee, Dong-Ki Hong, Kook-Nyung Lee, Hyun C. Yoon

**Affiliations:** 1 Department of Molecular Science & Technology, Ajou University, Suwon 16499, Korea; kkr4649@ajou.ac.kr (K.R.K.); Han.Yong@mayo.edu (Y.D.H.); moogoosla@ajou.ac.kr (H.J.C.); ursus780@ajou.ac.kr (K.W.L.); 2 Korea Electronics Technology Institute, Seongnam 13509, Korea; hdk0816@gmail.com (D.-K.H.); plummy@keti.re.kr (K.-N.L.)

**Keywords:** europium chelate, luminophore-encapsulated nanoparticle, time-resolved luminescence, immunosensing, cardiac troponin I

## Abstract

The use of a robust optical signaling probe with a high signal-to-noise ratio is important in the development of immunoassays. Lanthanide chelates are a promising material for this purpose, which provide time-resolved luminescence (TRL) due to their large Stokes shift and long luminescence lifetime. From this, they have attracted considerable interest in the in vitro diagnostics field. However, the direct use of lanthanide chelates is limited because their luminescent signal can be easily affected by various quenchers. To overcome this drawback, strategies that rely on the entrapment of lanthanide chelates inside nanoparticles, thereby enabling the protection of the lanthanide chelate from water, have been reported. However, the poor stability of the lanthanide-entrapped nanoparticles results in a significant fluctuation in TRL signal intensity, and this still remains a challenging issue. To address this, we have developed a Lanthanide chelate-Encapsulated Silica Nano Particle (LESNP) as a new immunosensing probe. In this approach, the lanthanide chelate is covalently crosslinked within the silane monomer during the silica nanoparticle formation. The resulting LESNP is physically stable and retains TRL properties of the parent lanthanide chelate. Using the probe, a highly sensitive, sandwich-based TRL immunoassay for the cardiac troponin I was conducted, exhibiting a limit of detection of 48 pg/mL. On the basis of the features of the LESNP such as TRL signaling capability, stability, and the ease of biofunctionalization, we expect that the LESNP can be widely applied in the development of TRL-based immunosensing.

## 1. Introduction

The sensitive and accurate immunoassay of disease markers in biological specimens from patients is a cornerstone in in vitro diagnostics (IVD) [1,2,3]. Especially, when the accuracy of the initial diagnosis is closely related to the survival rate of the patient, as is the case for acute myocardial infarction (AMI), it is very important to employ an effective analytical tool that enables the precise quantification of minute changes in concentration of the target analyte [4,5,6,7]. Early-stage analysis in disease is also important because it can help clinicians make appropriate clinical decisions to allow for prompt and effective treatment. From this point of view, the development of immunosensing technologies that provide high sensitivity and accuracy is regarded as the most significant issue in both academia and the IVD industry [1,4,7]. The sensitivity of any immunosensors is closely related to the type of signal reporter employed and its corresponding signaling capability [8,9,10]. In this regard, there have been numerous studies aimed at the development of materials that could be used as effective immunosensing probes [11,12,13,14]. A major strategy adopted in the development of a signaling label has been to focus on increasing the signal-to-noise ratio, thereby enabling the distinct separation of a target-specific signal from nonspecific signals. In particular, fluorescent materials (e.g., organic fluorescent dyes, fluorescent nanoparticles, and luminescent semiconductor nanocrystals), which exemplify the fluorescence immunoassay principle, have been widely used, since they provide robust and target-derived optical signals that are relatively easy to detect. However, the approach that employs the use of a signal label with a strong signal intensity is not sufficient to realize the goal of ultra-sensitive immunosensing [15,16,17,18,19,20]. In the practice of an ultra-sensitive assay, the signal from a target analyte on the left side of detection window will be very small. Therefore, the minimization and suppression of the background signal that interferes with the valid target-derived signal should be accomplished simultaneously [21,22]. With this in mind, the use of conventional fluorophores in an immuno-analysis that requires high sensitivity, such as the detection of cardiac troponin I (cTnI) for AMI diagnosis, is not sufficient [23,24,25,26]. From the viewpoint of fluorescence yield, the fluorophore Stokes shift, which is defined as the difference between the maximum absorption and emission wavelengths, should be large enough to prevent the reduction of fluorescence intensity caused by the overlap between the absorption spectrum and the emission spectrum [23,24,25]. Along with the Stoke shift, the fluorescence lifetime is one of the critical values in the suppression of background signal in a fluorescence immunoassay. Especially the auto-fluorescence, which originates from the sensing surface (e.g., nitrocellulose membrane or a polymeric substrate) as well as from fluorescent biomolecules present in the specimen (e.g., coenzymes and proteins), is regarded as candidates of signal interference. Although the intensity of auto-fluorescence is usually weak, it represents a significant background signal that cannot be ignored, especially for an ultra-sensitive immunoassay. To find a strategy that enables the elimination of the adverse effect of auto-fluorescence, researchers have focused on the short lifetime of auto-fluorescence [22,24]. Because auto-fluorescence generates and then disappears quickly, the use of a novel fluorophore that exhibits a longer lifetime is a promising solution to this issue. Therefore, the development of fluorophores exhibiting a large Stoke shift with a long lifetime is a desirable goal that could replace conventional fluorophores [25,27,28,29].

In this regard, the time-resolved luminescence (TRL) immunoassay utilizing lanthanide-chelates as the optical probe has attracted significant attention. Compared to that of a conventional immunoassay, the most distinctive feature is the use of lanthanide-chelate luminophores as the signaling probe. These probes have an extremely long luminescence lifetime (μs to ms range) compared to conventional fluorophores (ns range) [30,31,32]. Typically, the lanthanide-chelate luminophore is comprised of three main parts, including the lanthanide ion, the chelating group holding the lanthanide ion through a coordination bonding, and the antenna group enabling the transfer of excitation energy to the lanthanide ion [33,34]. Based on the matched electron energy states created by the coordination of the antenna group with the lanthanide ion, the absorbed excitation energy can be transferred to the lanthanide ion [35]. The lanthanide emission can be maintained for a long time due to the long-lasting excited state of the lanthanide ion and the inherent energy transfer between excited state and emission states (5D0 → 7FJ) [36]. From the extended luminescence lifetime, the target-specific luminescence signal induced from the lanthanide probe can be detected after the auto-fluorescence signal has completely disappeared. Therefore, the TRL assay method using lanthanide probes enables the development of ultra-sensitive immunoassays.

**Figure 1:**
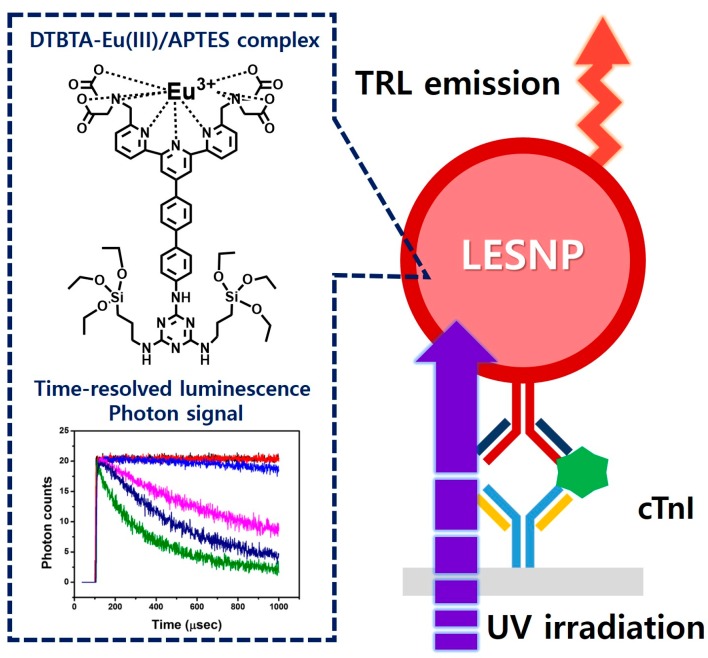
Schematic illustration of the TRL-based immunosensing using the developed LESNP probe. Using the TRL property of the europium-chelate, which was covalently encapsulated within the silica nanoparticle, the synthesized LESNP can provide stable TRL signals for the immunosensing of cardiac troponin I (cTnI).

However, the direct use of lanthanide chelates in the aqueous phase is hard to achieve because the luminescence from lanthanide is subject to interference and easily quenched by water and oxygen molecules [35]. Therefore, to protect luminophores from the attack by water and oxygen, the lanthanide chelates can be encapsulated in particulate forms. Up until now, a number of studies have reported the use of polymeric and silica materials for the encapsulation of lanthanide chelates [37,38,39]. Because these studies have adopted the strategy of simply impregnating the lanthanide chelates inside solid particles, there still exists the possibility of lanthanide leakage, followed by subsequent luminescence quenching [40]. Therefore, a method that enables the stable encapsulation of the lanthanide chelates inside a solid particle is required. Here, we report the development of a novel TRL-immunosensing probe comprised of a lanthanide chelate-encapsulated silica nanoparticle (LESNP) that exhibits a high TRL signal even under external physical stresses. To synthesize LESNP, we first synthesized a lanthanide chelate-silane complex that can act as a silica precursor and participate in the nanoparticle assembly. This complex was formed by conjugating the amino silane compound with the lanthanide chelate. During the synthesis of silica nanoparticles, the lanthanide chelate-silane complex became covalently crosslinked with the silica precursors and fixed inside the nanoparticle. Using this encapsulation approach, the lanthanide chelates can be retained in the LESNP without leakage, thereby allowing for the generation of stable TRL signals. In the present study, the mechanical structure, composition, stability against physical stress, and optical properties of this LESNP were determined using spectroscopic and microscopic approaches. Finally, to demonstrate the applicability of this newly developed LESNP as a signaling label for a TRL immunoassay, a sandwich-type immunosensing for cTnI was carried out (Figure 1). Details are reported herein.

## 2. Materials and Methods

### 2.1. Reagent and Apparatus

3-aminopropyl triethoxysilane (APTES), tetraethyl orthosilicate (TEOS), and succinic anhydride were acquired from Sigma Aldrich (St. Louis, MO, USA). Cyanuric chloride and sodium [4′-(4′-Amino-4-biphenylyl)-2,2′:6′,2′′-terpyridine-6,6′′-diylbis(methyliminodiacetato)]europate(III) (ATBTA-Eu^3+^) were purchased from TCI (Tokyo, Japan). Cyclohexane, 1-hexanol, Triton^TM^ X-100 (TX-100), Tween^®^-20, ethanolamine, ammonium hydroxide solution (28%), and glutaraldehyde (25%) were purchased from Sigma-Aldrich. Goat-anti-mouse IgG was purchased from Sigma-Aldrich. Mouse monoclonal anti-cardiac troponin I antibodies (clones 19C7 and 16A11) and the recombinant human cardiac troponin I antigen were purchased from Hytest (Turku, Finland). 1-ethyl-3-(3-dimethylaminopropyl) carbodiimide hydrochloride (EDC) and *N*-hydroxysulfosuccinimide (sulfo-NHS) were purchased from ThermoFisher (Waltham, MA, USA). Sulfo-NHS acetate was obtained from Pierce (Rockford, IL, USA). Sodium phosphate-buffered saline solution (0.1 M) containing 0.15 M NaCl (PBS, pH 7.2), 0.1 M 2-(n-morpholino)ethanesulfonic acid buffer solution (MES, pH 5.5), 0.1 M sodium bicarbonate buffer solution (pH 9.0 and pH 11.0), and 0.1 M sodium acetate buffer solution (pH 4.9) were prepared using doubly distilled and deionized water (DDW, specific resistance >18 MΩ·cm). For the dispersion and storage of the synthesized nanoparticle, a particle storage buffer solution from Ademtech (Pessac, France) was used. As a washing buffer solution for immunoassay, PBSTB was prepared by dissolving 0.05% Tween-20 and 1% bovine serum albumin (BSA) in 0.1 M PBS (pH 7.4).

### 2.2. Preparation of Europium-Chelates/Aminosilane Complex

As a source of europium-chelate luminophore allowing time-resolved luminescence signaling, ATBTA-Eu^3+^ was purchased and used for the preparation of the lanthanide chelate-encapsulated silica nanoparticle [30]. To covalently entrap the ATBTA-Eu^3+^ molecules into silica nanoparticles, the ATBTA-Eu^3+^ was modified to tag silane substances on europium chelate. Nishioka [31] previously reported the protocol for the functionalization of amine moiety in the europium chelate (ATBTA-Eu^3+^) to another amine-reactive group. First, 40 mM ATBTA-Eu^3+^ (ATBTA-europium chelate, AEC) and 54 mM cyanuric chloride were prepared in 0.1 M acetate buffer (pH 4.9) and acetone, respectively. Under a dark condition, 25 μL of cyanuric chloride was added to 60 μL of the AEC solution. The mixture was allowed to react for 30 min with constant mixing at room temperature. During this procedure, an intermediate compound, {2,2′,2′′,2′′′-{4′-{[(4,6-dichloro-1,3,5-triazin-2-yl)amino]biphenyl-4-yl}-2,2′:6′,2′′-terpyridine-6,6′′-diyl}bis-(methylenenitrilo)}tetrakis(acetato)} europium(III) (DTBTA-Eu^3+^, DEC), was formed as a pale yellow precipitate through the covalent conjugation of the cyanuric chloride with the amine moiety from AEC. After the conjugation procedure, DEC was collected by centrifugation and washed twice with acetone. The collected DEC precipitate was dried in a vacuum for 3 hours at room temperature and dissolved in 0.1 M bicarbonate buffer (pH 9.0). Then, 1.78 μL of 99% APTES was reacted with 1 mL of DEC (2 mM) for 150 min. After silane conjugation, reactants were stored in freezer. With the silane alkoside group from DTBTA-Eu^3+^/APTES complex (DEAC), it can be crosslinked with silica precursors (e.g., TEOS) to form nanoparticles [41,42].

### 2.3. Synthesis of Lanthanide Chelate-Encapsulated Silica Nanoparticles 

**Figure 2:**
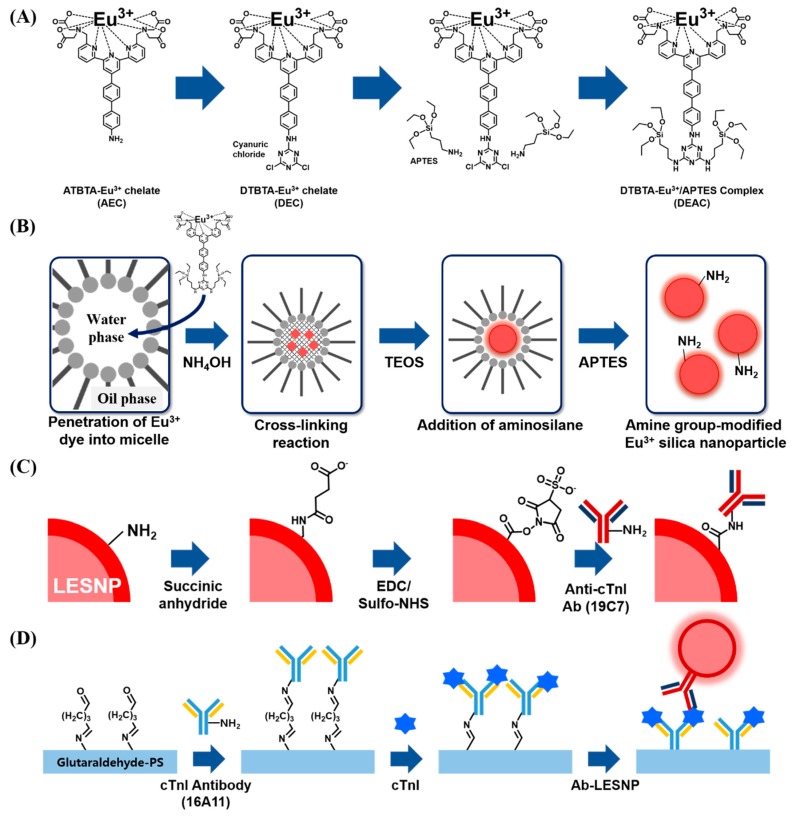
Schematic illustration of the procedures used for the synthesis of LESNPs, bio-conjugation of LESNPs, and the cTnI immunoassay using LESNPs. (**A**) procedure for the synthesis of the DTBTA-Eu^3+^/APTES complex (DEAC) precursor; (**B**) schematic illustration of the LESNP synthetic process; (**C**) illustration of the procedures for the surface modification of LESNP and antibody conjugation to LESNP; (**D**) cTnI sandwich immunoassay procedure using the LESNP probe.

Using the prepared DEAC as a europium chelate, the lanthanide chelate-encapsulated silica nanoparticles (LESNP) were synthesized by a water-in-oil (*w/o*) reverse micro-emulsion method, which is widely utilized in silica nanoparticle synthesis (Figure 2B) [43,44]. To prepare the oil phase of the micro-emulsion mixture, 4 mL of cyclohexane, 1 mL of 1-hexanol, and 1 mL of TX-100 were mixed. The water phase was prepared by mixing 295 μL of 2 mM DEAC solution with 35 μL of ammonium hydroxide (28%). To prepare the micro-emulsion containing uniform micelles, the prepared oil phase solution (6 mL) and water phase solution (0.33 mL) were mixed and vigorously stirred for 30 min at room temperature. Following this, 100 μL of TEOS was added to the prepared micro-emulsion solution to initiate the hydrolysis and condensation between the silane alkoxide group in TEOS and DEAC [42]. The reaction was allowed to proceed for 18 h at room temperature. Through this procedure, the silica precursors (TEOS) became infiltrated into the water-phase of the micelle containing the silane-terminated europium chelate complexes (DEAC). Consequently, the hydrolyzed silane alkoxide groups in DEAC and TEOS became crosslinked in the water-phase of the micelle, forming LESNPs (diameter ca. 100 nm). At the end of the reaction, 40 μL of APTES was then added to the solution to coat the surface of LESNPs to expose the amine functionality, which can be employed as the ligands for further bioconjugation with proteins (Figure 2B). After reacting for 6 h, the particle synthesis and surface modification reaction was terminated by adding an excess of acetone to the micro-emulsion [45]. The synthesized amine-terminated LESNPs were collected by centrifugation (2500 g) and sequentially washed with ethanol and distilled water.

### 2.4. Antibody Conjugation on Synthesized Amine Group-Terminated LESNPs

To prepare the LESNP-based immunoassay capable of detecting cTnI, a monoclonal antibody to cTnI (19C7 clone) was covalently conjugated to the surface of amine-terminated LESNPs by carboxylation followed by the EDC/NHS coupling reaction (Figure 2C) [46]. Prior to the conjugation procedure, the amine-terminated LESNPs were dispersed in 0.1 M carbonate buffer (pH 11) at 0.1% (*w/v*). To introduce carboxyl groups to the surface of the amine-modified LESNP, the LESNP suspension (0.1%) and 200 mM succinic anhydride solution (in DDW) were mixed at a 1:1 volume ratio with vigorous stirring for 5 h at room temperature [47,48]. As a result of this step, the amine groups on the LESNP surface are converted into carboxyl groups. Then, the carboxylated LESNP particles were collected by centrifugation for 30 min at 12,000 g and washed three times with PBS. After washing, the LESNPs were recovered from the PBS solution and stored in the dark at room temperature. To block any unreacted amine residues on the carboxylated LESNPs, sulfo-NHS acetate in PBS (10 mM) was then added to the LESNPs and allowed to react for 2 h in the dark. After washing the LESNP particles with PBS, the resulting LESNPs were re-suspended in 0.1 M MES buffer (pH 5.5) at 0.1% (*w/v*). Prior to activating the carboxyl groups on the LESNP using the EDC/NHS coupling reaction, solutions of 1 mg/mL of EDC and 1.7 mg/mL of sulfo-NHS were prepared in MES buffer. EDC solution (50 μL) and sulfo-NHS solution (50 μL) were then added to the LESNP suspension with stirring and reacted for 15 min in the dark. Immediately following this, 200 μL of cTnI detection antibody (19C7 clone, 150 μg/mL in PBS) was added to the activated LESNP suspension with gentle stirring for 1 h. To inactivate the unreacted sulfo-NHS groups on LESNPs, a mixture containing 20 mM of ethanolamine, 1% BSA and 0.01% PEG (*m/w* 3400) was added and reacted for 3 h. After this blocking procedure was completed, the antibody-conjugated LESNPs were washed three times with PBS and re-suspended in an Ademtech storage buffer solution at 4 °C [49].

### 2.5. Conjugation of the Surface Modification of LESNP

To confirm that the surface modification of LESNP had been accomplished as intended, fluorescence microscopic analysis was conducted. Prior to the test, three different types of LESNP samples having different surface structures, namely amine-terminated LESNP, carboxylated LESNP, and mouse IgG-modified LESNP, were prepared. For the observation of luminescence with the different LESNP samples, several types of base surfaces were also prepared, including an amine-terminated surface, a carboxylate-terminated surface, a BSA-passivated surface, and an anti-mouse IgG-modified surface. To construct these surfaces, a thin gold film-deposited silicon wafer was utilized as the starting substrate. Using a sputter system, Ti (50 nm) and Au (200 nm) layers were sequentially deposited onto the silicon wafer. The gold film-deposited silicon wafer was then diced into rectangular chips (2 cm × 1 cm). To remove impurities on the gold surface, the chips were cleaned by immersing them in piranha solution (1:4, H_2_O_2_:H_2_SO_4_) for 5 min. After rinsing with DDW, surface modification procedures based on the self-assembled monolayer technique were followed. For the construction of the amine-terminated surface, the gold chip was immersed in a 5 mM cystamine solution for 2 h [50]. Similarly, fabrications of the carboxylated surface and the amine-reactive surface were accomplished by immersing gold chips into 10 mM mercaptoundecanoic acid (MUA) solution (in ethanol) [51] and 5 mM 3-3′-dithiobis-propionic acid N-hydroxysuccinimide ester (DTSP) solution (in dimethyl sulfoxide) [52], respectively. The BSA-passivated surface was made by applying 1% BSA and 10 mM ethanolamine to the DTSP-modified gold surface. In a similar manner, the antibody-modified surface was prepared by adding an anti-mouse IgG (150 μg/mL) solution to the DTSP-modified surface.

To verify that the amine group was functionalized on the surface of the synthesized LESNP, the amine-terminated LESNP, which was collected right after the synthetic procedure, was applied to the amine-reactive gold surface. The BSA-inactivated surface was used as a negative control. To demonstrate that the carboxyl groups were successfully modified on the LESNP nanoparticle after succinic anhydride treatment, carboxylated LESNPs were incubated with the positively-charged amine-functionalized surface. A negatively-charged MUA-modified gold surface was used as a negative control. Finally, to confirm the successful coupling of the antibody to the LESNP by EDC/NHS coupling reaction, the mouse IgG-modified LESNPs were applied to the anti-mouse IgG-modified gold surface. The same LESNPs were incubated with the BSA-inactivated gold surface as a negative control. After allowing particle-surface interaction for 15 min, each surface was rinsed with PBSTB. Images of the resulting surfaces were analyzed using fluorescence microscopy with a 340-nm excitation filter and a 615-nm bandpass emission filter.

### 2.6. Construction of the cTnI Immunosensing Surface

For the setup of the sandwich type immunosensing assay for cTnI, the cTnI capture antibody (16A11 clone) was covalently immobilized on a chemically-modified transparent polystyrene (PS) surface as shown in Figure 2D [53,54,55]. Prior to the antibody conjugation, the PS surface was activated by using atmospheric pressure plasma treatment. The activated PS substrate was then immersed in a 10% APTES solution for 30 min. During the process, amine groups were developed on the activated PS surface through a covalent coating by APTES. After sequential washing with isopropyl alcohol and DDW, the APTES-coated PS substrate was dried using N_2_ gas. Next, the amine-terminated PS surface was covered with a punched (diameter = 4 mm) transparent silicon rubber sheet (thickness = 200 μm). This hole was utilized as a reaction well for the sandwich type cTnI immunoassay. To conjugate the cTnI antibody to the amine-terminated PS surface, 1% glutaraldehyde was added to the exposed PS surface for 1 h. After rinsing three times with PBS, 150 μg/mL of cTnI capturing antibody (16A11 clone, in PBS) was applied for 1 h and then the surface was washed. During this process, the cTnI capture antibody becomes covalently immobilized on the PS surface via imine bond formation between amine residues present on the antibody and the aldehyde group present on the PS surface. To block the unreacted aldehyde groups on the antibody-modified PS surface, 20 mM ethanolamine and 2% BSA (in PBS) were then sequentially added to the surface for 30 min each. After the blocking step, the resulting immuno-sensing surface was filled with PBS and stored in the dark at 4 °C until use.

### 2.7. Fabrication of the Homemade Time-Resolved Luminescence Analyzer

For the analysis of TRL signals from the LESNPs in the cTnI immunosensing, we constructed a bench-top TRL signal analyzer as shown in Figure S1 (see supplementary materials). As a light source to induce lanthanide-based luminescence from the LESNPs, a light guide-mounted 340-nm ultraviolet light-emitting diode (UV-LED, 50 mW) was utilized. As a photon detector, an emission filter (610–620 nm) mounted photomultiplier tube (PMT) was employed and installed above the UV-LED. The sample zone for the cTnI TRL sensing surface was located between the UV-LED and the PMT. The process of the TRL reading was as follows. First, an excitation light from the UV-LED is radiated toward the immuno-sensing surface containing the LESNPs through a light guide. After stimulation with UV light, the LESNPs generate a luminescence signal that reaches the PMT after passing through the emission filter. To acquire a TRL signal from the LESNP, time-gated control of the light source and the detector is required. To address this, a timing circuit that could precisely control both the light source and the optical receiver was constructed and connected with the UV-LED and the PMT. By adjusting the timing circuit, the on/off operation interval of the UV-LED and the photon counting interval of the PMT were selected [56]. The TRL analyzer was then connected to a computer and controlled by its own software, which was coded using the LabVIEW program (National Instruments, Austin, TX, USA). For practical TRL immunosensing of cTnI, the UV-LED was turned on for 100 μs and then turned off for the next 900 μs. After UV-LED operation, the PMT starts to count the photon signals from immunosensing surface for 900 μs immediately following the off signal from the UV-LED. This whole process was repeated 20 times per second. The TRL control software was programmed to integrate the photon signals obtained and then to provide averaged values.

### 2.8. TRL Immunosensing of cTnI Using LESNP

To demonstrate the performance of the developed LESNP probe on the TRL analyzer, a sandwich-type immunosensing capable of detecting cTnI was conducted. Prior to the assay, cTnI samples at various concentrations (0, 0.05, 0.5, 1.0, 5.0, and 20 ng/mL) were prepared in PBS (pH 7.4). Each sample was applied to the prepared cTnI immunosensing surface for 15 min. Then, the signaling antibody (19C7 clone)-conjugated LESNPs were applied to the sensing surface and allowed to react for 20 min and rinsed with PBSTB. The time-resolved luminescence photon signals from LESNPs, which represent LESNPs remaining on the cTnI immunosensing surface, were registered from the TRL analyzer. The cTnI immunoassays with different samples were repeated to obtain a calibration curve.

## 3. Results and Discussion

### 3.1. Synthesis of Lanthanide Chelate-Encapsulated Silica Nanoparticles

To develop luminescent nanoparticular probes exhibiting robust lanthanide-based TRL property, the lanthanide luminophore chelates should be encapsulated inside a solid particle to separate and protect the luminophore chelates from water molecules, which significantly reduce the luminescent signal by quenching [35]. In the present study, silica nanoparticles were employed as carriers to encapsulate a lanthanide luminophore. This nanoparticle was chosen for its facile synthesis and ease of further bioconjugation via silane chemistry. We anticipated that the high mechanical stability of silica would be effective at protecting and retaining the luminophore from external stresses. However, since a simple entrapment of luminophores inside a silica particle may still allow for leakage of the entrapped chemicals, a new approach to firmly encapsulate the lanthanide luminophore within the silica nanoparticle was required. To address this, we focused on the development of a nanoparticle synthetic protocol that allowed for the covalent conjugation of a lanthanide luminophore inside of a silica nanoparticle [56,57]. In our approach, ATBTA-Eu^3+^ (ATBTA-europium chelate, AEC) was employed as the lanthanide-chelate luminophore, since it contains the essential functional groups required for TRL signaling, a chelation group capable of coordinating the europium ion, and an antenna group that allows for the transfer of excitation light to the europium ion, as shown in Figure 2A [30]. 

**Figure 3:**
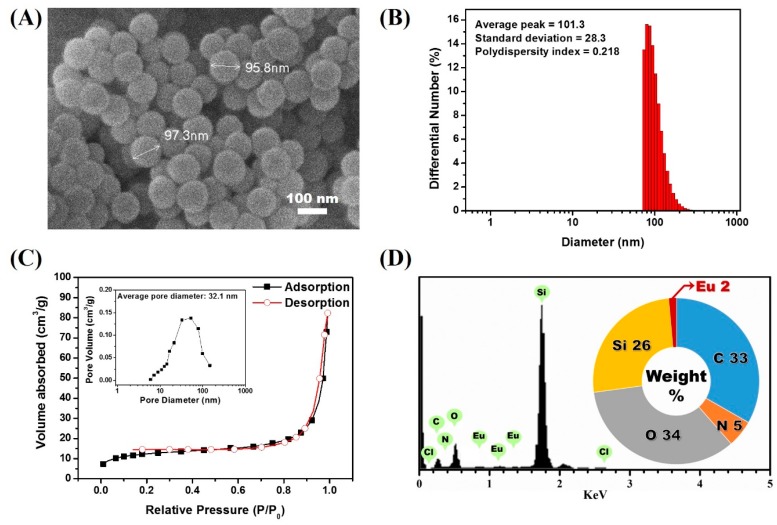
(**A**) scanning electron microscope (SEM) image of the LESNP; (**B**) average hydrodynamic diameter analysis result of LESNP; (**C**) nitrogen adsorption–desorption isotherm curve and its pore size distributions of prepared LESNP (inset); (**D**) EDX analysis result of the LESNP.

Another desirable feature of AEC is the existence of an additional amine group, which allows for easy chemical modification. As a first step, we elected to synthesize a europium luminophore-modified silica precursor compound that could be cross-linked during the silica nanoparticle synthetic process. To achieve this, we set out to conjugate the AEC with the silica precursor by following the previously reported protocol by Nishioka et al. Cyanuric chloride, a molecule containing three amine-reactive residues, was reacted with the AEC (Figure 2A) [58]. During the process, the amine-reactive residues in cyanuric chloride react with the amine moiety in the AEC, forming DTBTA-Eu^3+^ (DEC) [31]. Because the cyanuric chloride still contains two amine-reactive residues, which persist after the formation of DEC, further amine-targeting chemical modifications can be made. To conjugate the synthesized DEC with the silica precursor, an amine-terminated silane compound, APTES, was then reacted. In this step, DEC was conjugated with two APTES molecules as a result of the covalent interaction between the two amine-reactive sites from DEC and amine residues present in APTES, resulting in the generation of a DTBTA-Eu^3+^/APTES complex (DEAC). However, the DEAC preparation strategy could produce incomplete reactants such as single silane-conjugated DEC. There is also the possibility that an incomplete form of DEAC may participate in the particle generation. Although the incomplete reactants could be tethered to the surface of particles during the particle growing step, silane-conjugated luminophores could be encapsulated by silica precursors (TEOS) which is abundant in the oil phase of the microemulsion. With the introduction of the silane alkoxide residues, the DEAC can act as a silica precursor for the synthesis of silica nanoparticles [32]. Based on this, the lanthanide chelate-encapsulated silica nanoparticle (LESNP) was synthesized using the water-in-oil (*w/o*) reverse micro-emulsion method with DEAC as the precursor molecule (Figure 2B). In the water-in-oil reverse micro-emulsion method, micelles of a few hundred nanometers in diameter were produced. These micelles have a water phase inside, and each micelle acts as a nanometer-sized reactor to grow the silica nanoparticle [33,43]. For the LESNP synthesis, a primary silica precursor (TEOS) and DEAC were dispersed into the water phase, and accumulated on the inside of the micelle. In the presence of ammonium hydroxide, hydrolysis and condensation reactions occur between the silane alkoxide groups in TEOS and DEAC. During this LESNP growth, the presence of the APTES molecule, which is conjugated to the DEAC, allows the DEAC to become covalently crosslinked with other silica precursors. Because the LESNP was developed to be used as a probe in TRL-based immunoassay, it should contain functional groups for further conjugation with biomolecules such as antibodies. To introduce functional groups that allow for bioconjugation onto the LESNP surface, additional APTES was added to the micro-emulsion solution at the end of the LESNP growth reaction. As a result, the additional APTES penetrates inside of the micelle and caps the surface of the growing LESNPs through a condensation reaction. Since APTES contains an amine residue, the surface of the APTES-capped LESNP is therefore terminated with a high content of amine functional groups. The particle synthetic process was stopped by acetone, and the resulting amine-terminated LESNPs were collected by centrifugation. The scanning electron microscopy (SEM, JSM-6700F, JEOL) image in Figure 3A shows that the LESNPs were uniformly synthesized, having a spherical shape approximately 100 ± 10 nm in diameter. Given the uniformity in the size and spherical shape of the synthesized LESNPs, it can be assumed that the nanoparticle growth inside of the micelles, which act as nano-reactors for particle synthesis, works as expected.

The hydrodynamic diameter of LESNPs was also evaluated by dynamic light scattering (DLS, ELSZ-1000, Otuska, Osaka, Japan). As shown in Figure 3B, the average diameter of LESNP was found to be around 101 nm with 0.22 of polydispersity index. The polydispersity index value also indicates a small amount of aggregated LESNPs in solution. Furthermore, specific surface area and pore volume was verified with nitrogen adsorption and desorption isotherm analysis (Tristar 3000, Micromeritics, Norcross, GA, USA). Through the analysis, specific surface area and pore volume distribution was calculated by using the Brunauer–Emmett–Teller (BET) method and Barrett–Joyner–Halenda (BJH) methods. As depicted in Figure 3C, a nitrogen isotherm curve was corresponded to type II isotherm. From this, the calculated BET surface area and the total pore volume were 44.9 m^2^/g and 0.11 cm^3^/g, respectively. The type II isotherm represents a non-porous material with un-restricted monolayer-multilayer adsorption in nitrogen isotherm analysis [59]. The average pore size could be calculated from the BET area and the total pore volume was found to be as large as 32.1 nm (Figure 3C, inset). Considering the non-porous characteristic of LESNP, it can be expected that the calculated average pore size indicates the interstitial voids between particles [60]. 

To confirm that the europium ion was indeed encapsulated inside the LESNPs, an energy-dispersive X-ray spectroscopy (EDX) analysis was conducted. As shown in Figure 3D, distinct signal peaks corresponding to the europium ion were observed along with the silica peak. This result indicates that the europium chelates were crosslinked within the synthesized silica nanoparticles by the intended encapsulation strategy. After this confirmation of LESNP synthesis results, analyses of its optical properties were then carried out by both microscopic and spectroscopic methods.

### 3.2. Optical Property of the Synthesized LESNP

The optical characteristics of the synthesized LESNP were evaluated. First, to analyze the behavior of the luminescence signal from the LESNP, fluorescence microscopic tests were conducted. To be able to observe the luminescence signal from the europium-chelate, an additional optical filter unit having a 340-nm excitation filter and a 615-nm band-pass emission filter was installed on the fluorescence microscope [61]. 

**Figure 4:**
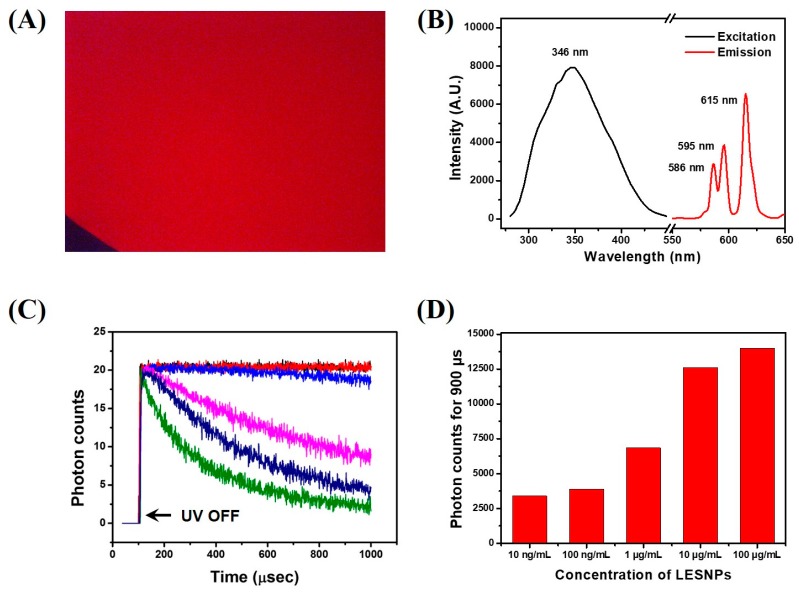
Optical property analysis of the LESNP. (**A**) fluorescence microscopy image of the LESNP in solution; (**B**) excitation and emission spectrum of the LESNP; (**C**) TRL photon counts curve, obtained using the LESNP at various concentrations (0–100 μg/mL); (**D**) integrated photon counts for the LESNP obtained at various concentrations (0.01–100 μg/mL).

Figure 4A shows that the LESNP dispersed in solution emits a bright red luminescent signal, which is similar to the typical luminescence signal of other europium chelates. For the spectral analysis of the luminescent signal, the LESNP sample was analyzed using a fluorescence spectrometer. As shown in Figure 4B, the LESNP exhibited a large Stokes shift. The maximum excitation wavelength was around 346 nm and the maximum emission wavelength was found around 615 nm under 340 nm excitation. In the emission spectrum, a number of 5D0 → 7FJ transition peaks were also observed due to the inherent luminescent process of the europium, with the peak around 615 nm exhibiting the strongest intensity [31]. On the basis of the spectral property of the LESNP, a home-made TRL analyzer that allowed for the photon counting of long-lived, LESNP-derived luminescence was employed (Figure S1). In the TRL analyzer, a 340 nm UV-LED was used as the light source and a 615 nm band-pass filter was used as the emission filter. The TRL property of LESNP was analyzed over a wide range of particle concentrations (0, 0.01, 0.1, 1, 10, and 100 μg/mL). The excitation light (UV-LED) was turned on for 100 μs and then turned off for 900 μs for TRL photon counting. After the UV-LED was turned-off, the PMT unit starts to count the long-lived luminescence photon signals from the LESNPs. As shown in Figure 4C, after the elimination of the excitation light, photon counts gradually decreased over the 900 μs signal registration time. As the concentration of LESNP increases, the change in photon counts during TRL analysis decreases. In Figure 4C, concentrated LESNP samples exhibited a saturated signal for the entire measurement time, which means that the photon count generated during the decaying process is more intense than the sensing capacity of the PMT. The PMT unit has a sensing capacity of 0–20 photons for one μsec. Compared to the saturated signal registration of high concentrations of LESNP, the TRL photon counts from the low concentrations of LESNP presented a serial decrease in photon counts. To determine the dose–response relationship between the LESNP concentration applied and the registered TRL photon counts, the total photon counts obtained over 900 μs timeframe were integrated and averaged. As shown in Figure 4D, the integrated photon count increased in proportion to the LESNP concentration. Based on the optical properties demonstrated from the tests, we conclude that the LESNP we have developed exhibits efficient and stable time-resolved luminescence characteristic that is suitable for use in a TRL-based immunoassay.

### 3.3. Confirmation of the Physical Stabtility of LESNPs

**Figure 5:**
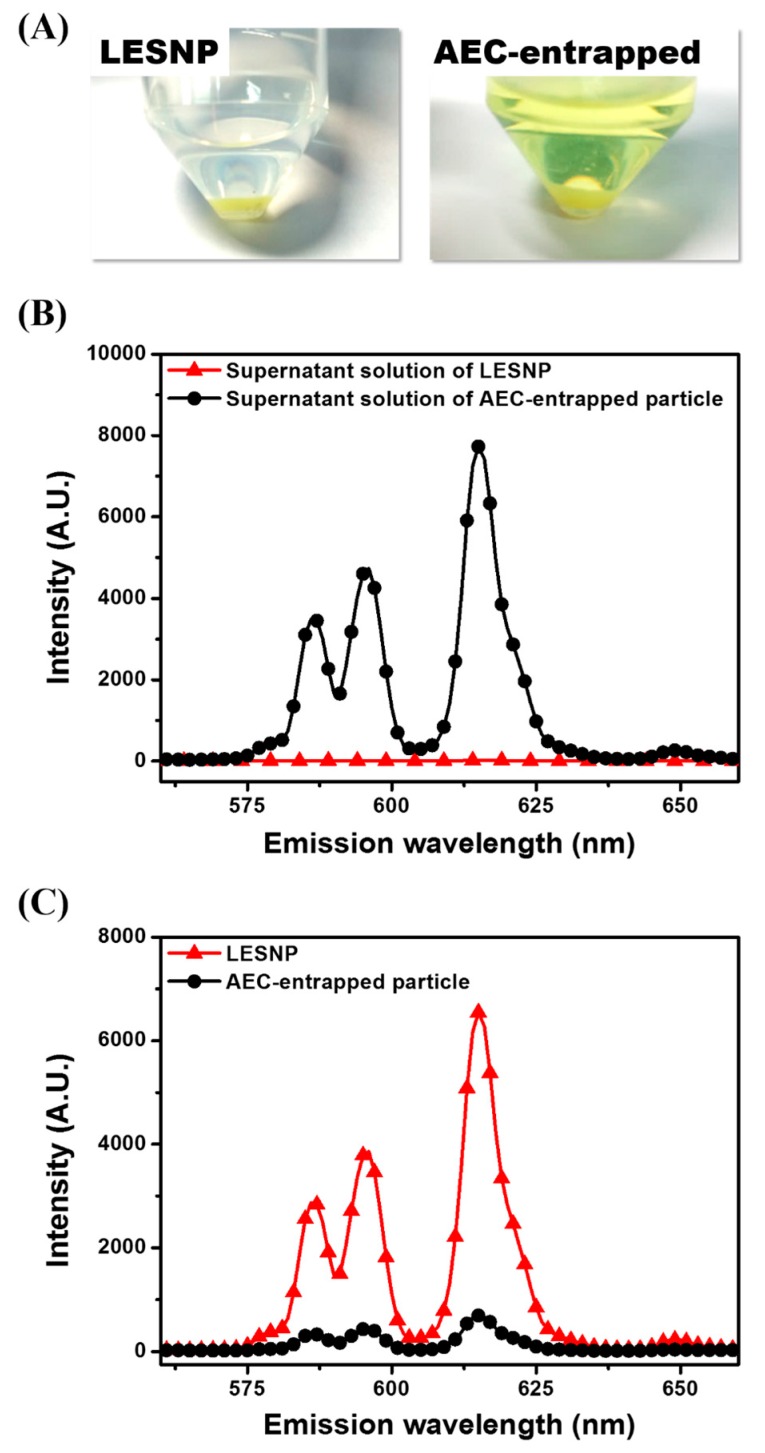
Comparative study comparing the behaviors of two different types of europium luminophore particles (LESNP and AEC-entrapped silica nanoparticle) following physical stress. (**A**) qualitative comparison of the LESNPs and the AEC-entrapped silica nanoparticles, after ultra-sonication and centrifugation; (**B**) the emission spectra of supernatant solutions from LESNPs and AEC-entrapped particles; (**C**) the emission spectra of centrifuged LESNPs and AEC-entrapped particles.

One of the main goals of this study is to develop a TRL probe that is physically stable at aqueous condition, preventing the potential leakage of luminophores from the particles [37,38,39]. To demonstrate the effectiveness of our approach, we evaluated the fate of encapsulated luminophore in the LESNP after applying a physical stress. A solution of LESNP was subjected to ultra-sonication for five minutes and the resulting LESNPs were collected and stored at 4 °C in the dark. After 1 h of storage, the particle fraction and supernatant solution were collected by centrifugation and their emission spectra were analyzed using a fluorescence spectrometer with a 340 nm UV light source. As a control, an AEC-entrapped silica nanoparticle (diameter = ca. 100 nm) was also prepared and analyzed in the same way. In the case of the control particle, the AEC was simply entrapped and not crosslinked inside the silica nanoparticle. It should also be noted that during the synthesis of the AEC-entrapped silica nanoparticle, we used the same amount of chemicals, including luminophores and silica precursors, as were employed during the LESNP synthesis. As shown in Figure 5A, the supernatant from the ultra-sonicated and centrifuged LESNP was clear and colorless, whereas the supernatant from the AEC-entrapped silica nanoparticle exhibited a distinct yellowish-green color. Considering that ATBTA-Eu^3+^ exhibits a yellowish-green color in solution, we thought that this color is from the leakage of ATBTA-Eu^3+^ from the AEC-entrapped particle to the solution phase. In contrast to the color seen in the supernatants, the pelleted LESNPs exhibited a much deeper green color than the pelleted AEC-entrapped silica nanoparticles, supporting the better stability of LESNP. From this comparison, we believe that the LESNP is more stable and could be utilized for an extended time even under stress conditions such as rigorous mixing and sonication. This qualitative analysis was then confirmed by spectroscopic analyses. As shown in Figure 5B, the supernatant from the AEC-entrapped solution showed an intense emission spectrum, which corresponded to the emission spectrum of ATBTA-Eu^3+^, whereas no emission spectrum was observed in the supernatant collected from the LESNPs. This result indicates that the luminophore was efficiently crosslinked with the silane precursors and therefore retained inside the LESNP even under a physical stress such as ultra-sonication. To quantify the retention of europium luminophore in the different particle types, the emission spectra of each particle were also analyzed as shown in Figure 5C. The intensity of the luminescence emission spectrum, indicating the amount of luminophore, was stronger for the LESNPs than for the AEC-entrapped particles. Taken together, the data confirms that the cross-linking strategy designed to retain the europium luminophore within the nanoparticle behaved as desired and was able to prevent luminophore leakage. On the basis of this observation, we believe that our synthetic strategy enables the preparation of a stable luminophore-containing nanoparticle.

### 3.4. Surface Modification of LESNPs for Biofunctionalization

**Figure 6:**
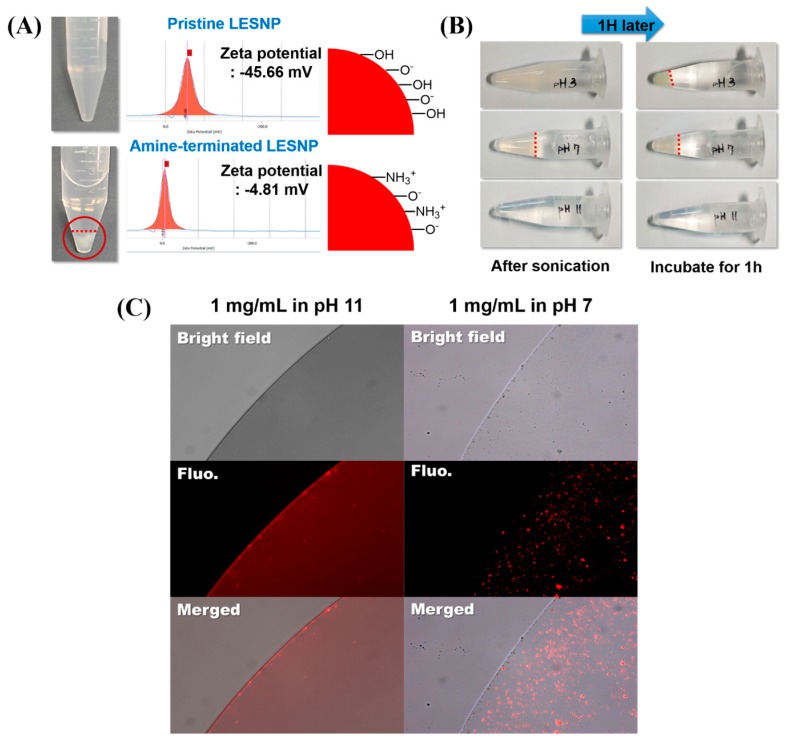
(**A**) comparison of the dispersion behaviors of the amine-terminated LESNP under different pH conditions. The upper panel shows the image of a pristine LESNP and its zeta-potential analysis. The lower panel shows the image of an amine-terminated LESNP and its zeta-potential analysis; (**B**) images of the amine-terminated LESNP in three different types of buffer solutions at different pHs (pH 3, 7, and 11). The aggregation of particles under the three different conditions was observed for one hour; (**C**) the result of a fluorescence microscopy analysis of the amine-terminated LESNP in two different buffer solutions of pH 7 and pH 11, respectively.

To employ the LESNP as a biosensing probe, the particle should be easily conjugated with biomolecules such as antibodies. To achieve the effective conjugation of an antibody to the particle, the particle in solution should be easily dispersed without aggregation [62]. We therefore conducted an optimization study for the surface modification of LESNP (Figure 6). Compared to the pristine LESNP (non-amine functionalized), which could be completely dispersed in DDW (pH 6.9), the amine-functionalized LESNP showed significant aggregation in DDW (Figure 6A). By analyzing the zeta-potential of the samples in this condition, it was revealed that this was due to the surface charge difference of the particles, as was depicted in the figure. In case of pristine LESNP, its zeta-potential was measured as −45.66 mV. This can be interpreted as the effect of the many surface hydroxyl groups, derived from the TEOS precursor having a negative charge at the given pH 6.9 [41,63]. Compared to this, the amine-functionalized LESNP, which was capped with APTES, exhibited a zeta-potential of −4.81 mV. Because amine-containing APTES was added in the synthesis of amine-terminated LESNP (see Figure 2B, vide supra), positively charged amine groups were introduced in the presence of negatively charged hydroxyl groups on the particle surface [41]. Therefore, there would exists charge compensation that causes the neutralization of net charge. Considering that particles could be completely dispersed when their zeta-potential was over ±30 mV, the neutralized surface charge (−4.81 mV) of the amine-functionalized LESNP would lead to particle aggregation by hydrophobic interactions [41,64]. With the result, we should find the optimum pH condition at which LESNP could be easily handled and conjugated to biomolecules without significant aggregation.

To determine solution condition, three different types of buffers with different pH were prepared including an acetate buffered solution (pH 3), a phosphate buffered solution (pH 7), and a carbonate buffered solution (pH 11). A sample of the amine-terminated LESNP was then dissolved in each buffer solution at a final concentration of 1 mg/mL. After 5 min of ultra-sonication, the particle dispersion was observed for 1 h at room temperature. As shown in Figure 6B, the particles in the pH 3 and pH 7 buffer solutions aggregated within 10 min. However, the LESNPs in the pH 11 buffer solution were completely dispersed in the solution phase without aggregation over an hour. This result was also confirmed by fluorescence microscopy observation. As shown in Figure 6C, large red-fluorescent dots were observed in the image of the LESNPs in the pH 7 buffer solution (right panel). The red fluorescent dots represent aggregated LESNPs considering particle size and magnification. In contrast, LESNPs in the pH 11 buffer solution exhibited a reddish luminescence that was homogeneously dispersed without aggregation throughout the solution (Figure 6C, left panel). We interpret this result as showing that under the alkaline conditions, the protonated amine group (–NH_3_^+^) becomes deprotonated to the neutral amine residue (–NH_2_), thereby reducing the total number of positive charges. This results in an increase in the total net negative charge, leading to a prevention of particle aggregation [64]. Based on this result, we concluded that a buffer solution of pH 11 should be used for particle storage and conjugation reactions with the LESNP. The amine-terminated LESNPs were stably dispersed in 0.1 M carbonate buffer (pH 11) at 0.1% (*w/v*) concentration.

### 3.5. Conjugation of Biomolecules on LESNP

The EDC/NHS coupling method was utilized to conjugate the antibody molecules to the LESNP. To achieve this, the carboxylation of the amine-functionalized LESNP was carried out by reacting the LESNP with succinic anhydride. In this reaction, succinic anhydride is reacted to the amine residues through a ring opening reaction resulting in the formation of a carboxyl group. Then, any remaining unreacted amine residues on LESNP were blocked by treating with sulfo-NHS acetate. Finally, by the EDC/NHS coupling reaction, the antibody was conjugated to the LESNP through amide bond formation.

**Figure 7:**
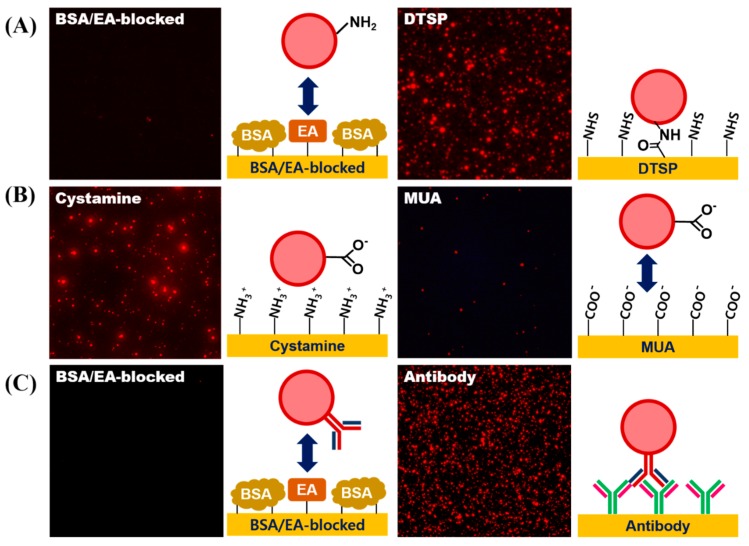
Verification of the surface modification processes for LESNP synthesis. (**A**) confirmation of successful amine-functionalization of the LESNP. To assess the presence of functional amine groups on the LESNPs, the amine-terminated LESNP was applied to an amine reactive DTSP-modified gold surface or a BSA-inactivated surface; (**B**) confirmation of successful carboxyl-functionalization of the LESNP. To assess the presence of carboxyl groups on the LESNP, the carboxylated LESNP was applied to an MUA-modified gold surface exhibiting a negative charge or to a cystamine-modified surface exhibiting a positive charge; (**C**) confirmation of successful antibody conjugation to the LESNP. To demonstrate the presence of conjugated antibody (mouse IgG) on the surface of the LESNP, the mouse IgG-conjugated LESNP was applied to either an anti-mouse IgG-immobilized surface or to a BSA-modified surface.

To demonstrate whether each surface modification step was accomplished successfully, a fluorescence microscopic analysis was conducted. To assess the introduction of the amine-functionality on the LESNP, the sample was reacted with an amine-reactive DTSP-modified gold surface, while a BSA-coated gold surface was used as a negative control. As shown in Figure 7A, the red fluorescent signal from the LESNP was only observed on the gold surface modified with the amine-reactive surface. This is because the amine-functionalized LESNP was immobilized onto the amine-reactive DTSP surface on the gold surface via amide bond formation. In contrast, the LESNP was rejected from the BSA-coated surface. These results indicate that the amine functionality was successfully made on the LESNP surface during the synthetic process. 

Next, to assess the introduction of functional carboxyl groups onto the LESNP, the carboxylated LESNP was prepared and reacted onto either a positively-charged surface (cystamine-modified gold surface) or a negatively-charged surface (MUA-modified gold surface). As shown in Figure 7B, a red fluorescent signal was only observed with the positively-charged cystamine surface whereas no fluorescent signal was observed with the negatively charged MUA surface. We consider that this confirms that the introduction of a negatively-charged carboxyl group onto the LESNP surface by succinic anhydride treatment has been successful. The carboxylated, negatively-charged LESNP readily binds to the positively-charged cystamine surface through an electrostatic interaction, while it does not interact with the negatively-charged MUA surface because of electrostatic repulsion. 

Finally, to demonstrate the antibody conjugation to the LESNP, the mouse IgG-conjugated LESNP was prepared and incubated with gold surfaces, which were modified to have either an anti-mouse IgG antibody or BSA, respectively. As shown in Figure 7C, a red fluorescent signal was only observed for the anti-mouse IgG-antibody modified gold surface, whereas no fluorescent signal was found on the BSA-immobilized surface. Considering that an immuno-affinity reaction only occurs between the mouse IgG and the anti-mouse IgG antibody biospecifically, the presence of luminescence only on the anti-mouse IgG-antibody modified surface can be interpreted as the signal from biospecifically bound LESNP. Therefore, we concluded that the antibody was successfully conjugated to the carboxylated LESNP by the EDC/NHS coupling method. Given the successful covalent coupling of the anti-cTnI antibody to the LESNP, we could next explore if this reagent could be used as an immunosensing probe in a TRL-based cTnI immunosensing.

### 3.6. TRL Immunoassay of cTnI Using Anti-cTnI IgG-Conjugated LESNP

**Figure 8:**
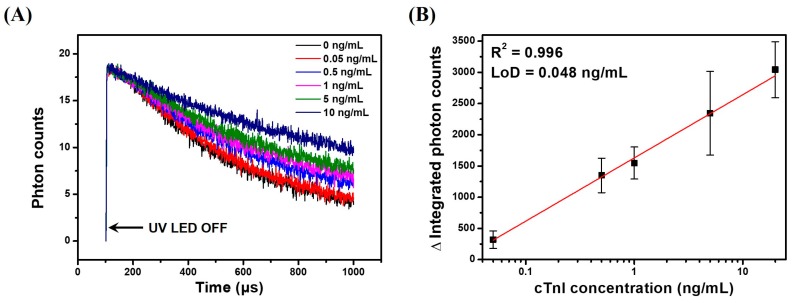
Results of the TRL–based cTnI immunoassay using the LESNP immunosensing probe and a home-made TRL analyzer. (**A**) the TRL photon counts curve for the cTnI samples at various concentrations (0, 0.05, 0.5, 1, 5, and 20 ng/mL); (**B**) calibration curve for the TRL-based cTnI sandwich-type immunoassay.

To demonstrate the applicability of the LESNP as a TRL-based assay probe, we conducted a sandwich-type immunoassay for cTnI, an AMI-specific biomarker, as a model immunosensing system. Before the assay, samples containing different concentrations of cTnI (0, 0.05, 0.5, 1, 5, and 20 ng/mL) were prepared in PBS. A cTnI capture antibody (clone 16A11) was covalently immobilized onto a transparent PS substrate to create an immunosensing surface. The cTnI samples with predetermined concentrations and the anti-cTnI antibody (clone 19C7)-modified LESNP were then sequentially applied to the immunosensing surface. After incubation, the cTnI immunosensing surface was then placed between the PMT module and UV-LED light source in the home-made TRL analyzer (Figure S1). The TRL photon signal generated from the cTnI immunosensing surface was then analyzed. As shown in Figure 8A, the integrated photon counts arising from the TRL emission by the LESNP present on the cTnI immunosensing surface gradually decreased over the 900 μs signal registration process. As expected, the rate of decrease in photon count was inversely proportional to the concentration of cTnI. This result indicates that the number of TRL photons emitted from the LESNP on the cTnI immunosensing surface increased proportionally to the concentration of cTnI in specimen.

To plot the calibration curve, the total TRL photon counts over 1 s of the signal registration process were integrated, and their triplicate experimental data was averaged. As shown in Figure 8B, the integrated photon counts increased linearly (R^2^ = 0.996) with the concentration of cTnI over a range from 50 pg/mL to 10 ng/mL. Using the Clinical and Laboratory Standard Institute (CLSI) guidelines, the limit of detection for TRL-based cTnI immunoassay was calculated to be around 48 pg/mL [65]. Currently, for the practical clinical application for AMI diagnosis, it is recommended that the detection limit of a cTnI immunoassay should be 50 pg/mL [66]. Therefore, the result suggests that TRL-based cTnI immunoassay can be used for early diagnosis of AMI, and it could meet the requirements for clinical application.

## 4. Conclusions

In this study, a lanthanide chelate-encapsulated silica nanoparticle (LESNP), having a stable and robust TRL signal, was developed as a TRL-based immunosensing probe. By crosslinking the europium-chelate luminophores with the silica precursors during nanoparticle synthesis, the europium luminophore could be firmly and covalently encapsulated inside the LESNP. The europium luminophore was stably retained inside the particle even under strong physical stress. The encapsulated europium-chelate luminophore within the LESNP had a large Stoke shift and good TRL characteristics, enabling suppression of background fluorescent signals. By employing LESNP, we successfully conducted a sandwich-type cTnI immunoassay for AMI. On the basis of this criteria, we believe that the LESNP reagent that we have developed has the potential to be used as a TRL immunosensing label for diagnostic assays that require high sensitivity and stability.

## Supplementary Materials

The following is available online at www.mdpi.com/2079-6374/7/4/48/s1, Figure S1: Constructed TRL analyzer (A) overall instrument layout of TRL analysis system; (B) schematic illustration of the structure of the developed TRL analyzer.
