# Supplementary material for: Encapsulation-Stabilized, Europium Containing Nanoparticle as a Probe for Time-Resolved luminescence Detection of Cardiac Troponin I
Source: Biosensors (Basel). 2017 Oct 18;7(4):48. doi: 10.3390/bios7040048 (PMC5746771; doi:10.3390/bios7040048)
Supplement: Supplementary file 1 [file biosensors-07-00048-s001.pdf]

biosensors

Supplementary Material: Encapsulation-Stabilized, Europium Containing Nanoparticle as a Probe for Time-Resolved luminescence Detection of Cardiac Troponin I

Ka Ram Kim [1,†], Yong Duk Han [1,†], Hyeong Jin Chun [1], Kyung Won Lee [1], Dong-Ki Hong [2], Kook-Nyung Lee [2] and Hyun C. Yoon [1,*]

[1] Department of Molecular Science & Technology, Ajou University, Suwon 16499, Korea; kkr4649@ajou.ac.kr (K.R.K.); Han.Yong@mayo.edu (Y.D.H.); moogoosla@ajou.ac.kr (H.J.C.); ursus780@ajou.ac.kr (K.W.L.)
[2] Korea Electronics Technology Institute, Seongnam 13509, Korea; hdk0816@gmail.com (D.-K.H.); plummy@keti.re.kr (K.-N.L.)
* Correspondence: hcyoon@ajou.ac.kr; Tel.: +82-31-219-2512
† These authors contributed equally.

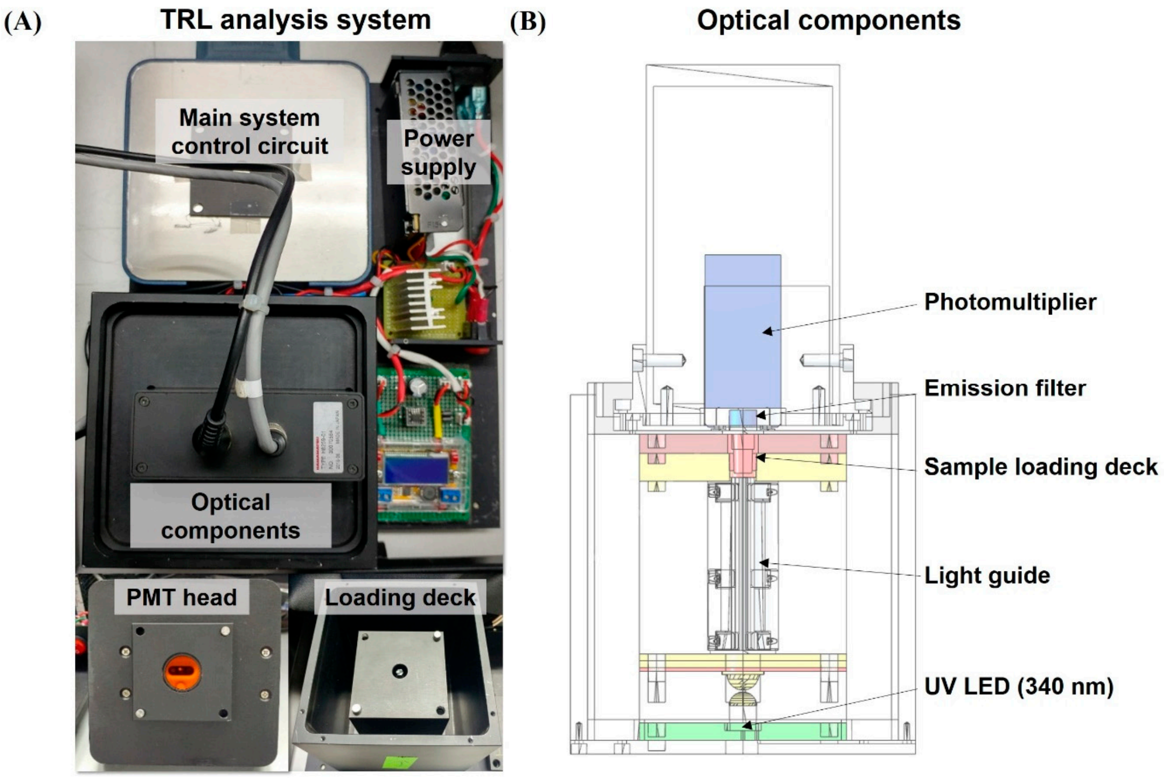

Figure S1. Constructed TRL analyzer (**A**) Overall instrument layout of TRL analysis system. (**B**) Schematic illustration of the structure of the developed TRL analyzer.
